# Supporting Multidisciplinary Analytic Skills: An Innovative Training Platform for Capacity Building

**DOI:** 10.23889/ijpds.v1i1.329

**Published:** 2017-04-19

**Authors:** Ann Greenwood, Maxine Reitsma

**Affiliations:** 1 Population Data BC; 2 University of Victoria, Division of Continuing Studies

## Objectives

To serve the emerging multidisciplinary skills and capacity building needs of Population Health Researchers within a rapidly diversifying field.

Population Health Research is inherently interdisciplinary, multifaceted and firmly rooted in the evolving connections between place, time and related socioeconomic processes. To excel in this rapidly diversifying field, individuals require a broad range of multidisciplinary skills. Supporting the development of these skills through innovative training platforms is one key way to build capacity for emerging 21 Century researchers and health professionals.

## Approach

Establishment of an innovative research training platform that supports skill development in a timely, collaborative and practiced based environment

The growing importance of data analytics and spatial thinking as it pertains to the worlds growing health concerns, be they social, physical or environmental - demands approaches that serve real time and remotely accessed, exploratory and highly collaborative research environments. A case example will be provided concerning a tri-party training platform that is serving the multidisciplinary skill requirements of new and mid-career population health professionals. Designed in collaboration with a tri-university research platform, the innovative, practice-based training environment both mirrors and supports many of the day to day skill development needs of health and social science researchers. 

## Results

The multidisciplinary focus of this specialized training platform is successfully addressing the skill development needs of a diverse cross section of health research professionals.

Trainees are bringing a wealth of experience and knowledge to share with their online colleagues, supporting a rich, practice based education and skills development environment. Those enrolled in the program possess backgrounds ranging from Population and Public Health, Epidemiology, Statistics and Sociology to Medicine to Psychology, Geography, Biostatistics and International Health.

## Conclusion

Providing timely, practical, hands-on analytic skills training is critical to building the capacity of new and mid-career researchers and health professionals.

Direct application of these new skills is an essential outcome and best measure of success. We are listening to our trainees and learning as we grow.

 Read what trainees are saying about our certificate courses https://www.popdata.bc.ca/etu/testimonials/PHDA

